# Protein-Protein Interaction Inhibitors Targeting the Eph-Ephrin System with a Focus on Amino Acid Conjugates of Bile Acids

**DOI:** 10.3390/ph15020137

**Published:** 2022-01-24

**Authors:** Lorenzo Guidetti, Riccardo Castelli, Laura Scalvini, Francesca Ferlenghi, Miriam Corrado, Carmine Giorgio, Massimiliano Tognolini, Alessio Lodola

**Affiliations:** Department of Food and Drug, University of Parma, I-43124 Parma, Italy; lorenzo.guidetti@unipr.it (L.G.); riccardo.castelli@unipr.it (R.C.); laura.scalvini@unipr.it (L.S.); francesca.ferlenghi@unipr.it (F.F.); miriam.corrado@unipr.it (M.C.); carmine.giorgio@unipr.it (C.G.)

**Keywords:** Eph receptor, ephrin, bile acids, protein-protein interaction, small molecules, antagonist, UniPR1331

## Abstract

The role of the Eph-ephrin system in the etiology of pathological conditions has been consolidated throughout the years. In this context, approaches directed against this signaling system, intended to modulate its activity, can be strategic therapeutic opportunities. Currently, the most promising class of compounds able to interfere with the Eph receptor-ephrin protein interaction is composed of synthetic derivatives of bile acids. In the present review, we summarize the progresses achieved, in terms of chemical expansions and structure-activity relationships, both in the steroidal core and the terminal carboxylic acid group, along with the pharmacological characterization for the most promising Eph-ephrin antagonists in in vivo settings.

## 1. Introduction

Erythropoietin-producing human hepatocellular (Eph) receptors form the largest subfamily of receptor tyrosine-kinases (RTKs), with several known members [1]. They are classified into two subclasses, A (EphA1-EphA8, EphA10) and B (EphB1-EphB4, EphB6) based on their amino acid sequences, as well as for their affinity for a class of membrane proteins, known as ephrins [2]. Eph receptors are composed by an extracellular region containing the ligand-binding domain (LBD) responsible for the interaction with the ephrins, a transmembrane portion, and a cytoplasmatic domain, which includes (i) a catalytically competent tyrosine-kinase domain; (ii) a sterile alpha motif (SAM); and (iii) a PDZ-binding motif [3], all dealing with signal transductions in Eph receptor expressing cells [4].

The ephrins are membrane proteins classified in two groups upon the sequence homology. The distinctive element between ephrin-A and -B is the presence in the latter one of a cytoplasmatic portion where a tyrosine-rich domain and a PDZ binding domain are present, while ephrin-As are exposed on the membrane by a glycosylphosphatidylinositol (GPI) anchor [4].

Due to their organization and localization, Eph-ephrin association occurs when contiguous cells have physical contact with one another [5]. Once a first Eph-ephrin heterodimer is formed, this event triggers the formation of heterotetramers; these structures associate further with generate oligomers [6]. It is now well understood that, upon Eph-ephrin association, Eph receptors form large polymeric clusters, and the phenotypic cellular response will depend on the size, composition, and spatiotemporal assembly [7,8].

A unique feature of the Eph-ephrin system is the ability to generate bidirectional signals [9]. The formation of an Eph-ephrin cluster generates forward signals in receptor expressing cells and reverse signals in ligand expressing cells [10,11,12]. The Eph-ephrin system plays a crucial role during embryogenesis as it is essential for development processes including cell migration and differentiation [10,13,14,15]. Conversely, in adults, the expression of this system is limited to those tissues where cell renewal is frequent, i.e., skin [16], breast [17], and intestinal tissues [18].

Compelling data gathered in the last two decades evidenced that the Eph-ephrin system is involved in the insurgence of different pathological conditions, including cancer diseases [19,20,21,22]. Its involvement within different facets of tumorigenesis and cancer progression, such as tumor angiogenesis, vasculomimicry, metastasis development, and tumor stem cell propagation, were reported and exhaustively described in the literature [20,21]. The Eph-ephrin system has emerged as an oncogenic pathway driving the insurgence of solid and liquid tumors. For instance, high expression of EphA2 and EphA3 has been linked to poor survival identified in glioblastoma multiforme (GBM) [23,24,25]. Similarly, EphB4, and ephrin-B2 have recently been found to concur to proliferation and in the invasive potential of GBM [26]. These and other findings indicate that multiple Eph receptors and ephrin ligand subtypes synergistically act in the insurgence of this type of cancer [27].

This scenario has therefore prompted the search of molecular agents targeting the Eph-ephrin system with a two-fold aim: (i) confirming the relevance of clinical data reported in the literature; and (ii) designing an efficient strategy based on biologics and small molecules to interact with targets of this elusive system in cancer therapy.

## 2. Agents Targeting the Eph-Eph System

### 2.1. Biologics

First attempts to target the Eph-ephrin system involved monoclonal antibodies (mAbs) and soluble forms of the Eph receptors or ephrin proteins. Monoclonal antibodies, acting in most cases as agonist agents, disrupt the Eph receptor ligand-independent activities mimicking the binding of the natural ligand at the extracellular LBD level. However, mAb agonists promote EphA2 degradation after induction of the phosphorylation activity of the receptor leading to an efficient blocking of forward signal transduction [28,29,30].

One of the most investigated mAb is MEDI-547, a fully human monoclonal antibody, identified after the phage display selection, which targets the LBD of EphA2 [31]. Its CDR-H_3_ loop mimics the interaction between EphA2 receptor–ephrin-A1, inducing the phosphorylation and the internalization of the receptor [32,33]. MEDI-547 reached the phase I clinical trial in patients with relapsed and refractory solid tumors (NCT00796055), but its safety and tolerability were not confirmed due to treatment-related bleeding and coagulation events [33]. Other recent mAbs that reached clinical phases are DS-8895, an anti-EphA2 monoclonal antibody developed by Daichii-Sankyo, and KB004, a non-fucosylated IgG1k antibody developed by Kalobios, and known by the name ifabotuzumab. The former, after a favorable phase I, was discontinued [34], while the latter reached phase II in patients with EphA3-expressing hematologic malignancies but discontinued for myelofibrosis (MF) (NCT01211691). KB004 is now under investigation in clinical phase I for glioblastoma multiforme (GBM) (NCT03374943) [35,36,37].

Soluble fragments of Eph receptors are alternative approaches to modulate Eph forward signaling. Receptors engineered with Fc domain of IgGs to increase their stability, can be used to compete with the endogenous Eph-ephrin system through ligand binding. The most advanced biological agent is a recombinant protein in which the ectodomain of EphB4 is fused with human serum albumin. A phase II trial study has been testing the combination of pembrolizumab and sEphB4-HSA in patients with previously treated urothelial carcinoma (NCT02717156) [38]. Another pilot study is focused on the administration of sEphB4-HSA before surgery in patients with bladder, prostate, or kidney cancer, with the aim to shrink the tumor and increase the amount of healthy tissue to be preserved with the surgery (NCT02767921) [39].

### 2.2. Peptides and Peptidomimetics

Another strategy to block Eph-ephrin signaling is to harness peptides of medium size. The mechanism of action of this class of agents is represented by the occupation of the LBD of Eph receptor, preventing the binding of the ephrin ligands.

**Figure 1:**
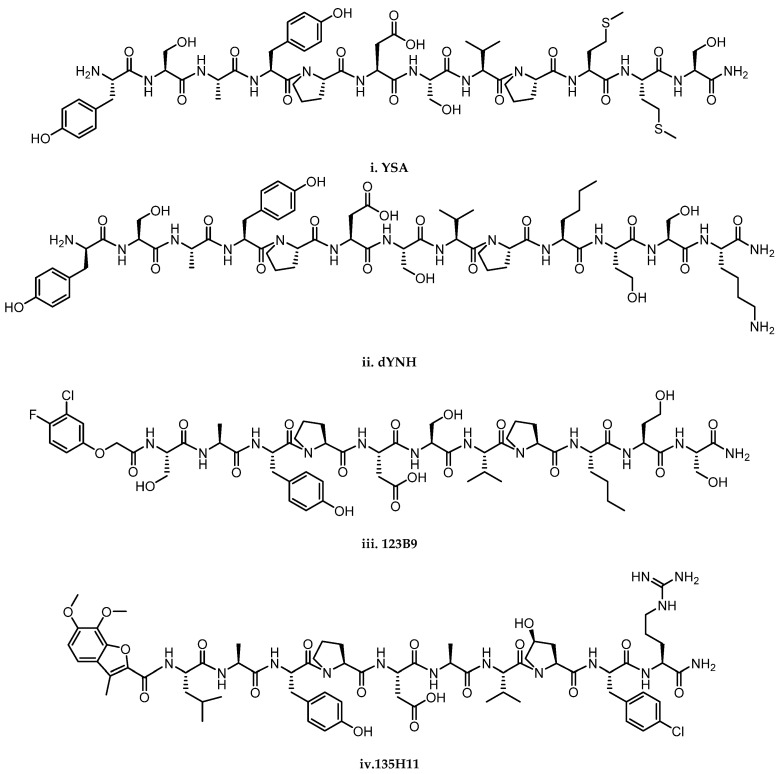
Structure of peptides and peptidomimetics antagonist of EphA2 receptor. **i**. YSA; **ii**. dNYH; **iii**. 123B9; **iv**. 135H11.

A first generation of peptides was identified by Pasquale and colleagues through a phage display screening. The approach led to the discovery of YSA and SWL (Figure 1i), two 12-mer peptides, showing high potency and selectivity for the EphA2 receptor. YSA and SWL display an agonist behavior, inducing receptor phosphorylation and signal transduction [40]. This and other studies allowed drawing a robust structure–activity relationship around peptides binding to Eph. For instance, fragments that preferentially bind class-A Eph receptors possess in their sequence a conserved Ω-X-X-Ω motif, where Ω represents an aromatic amino acid residue and X is a non-conserved amino acid. This binding motif is also present in the G-H loop of nearly all ephrin ligands, confirming the importance of Ω-X-X-Ω motif for Eph receptor binding [41]. APY, KYL, and VTM peptides were later reported as selective antagonists of EphA4 receptor able to prevent receptor activation [42]. While showing promising good pharmacodynamic properties, most of these peptides suffer from an extensive and rapid degradation in plasma. Modifications of these linear peptides, intended to overcome metabolic stability issues, were based on cyclization (i.e., through cysteine-cysteine disulfide bridges), PEGylation, dimerization, insertion of unnatural amino acids, or inclusion into nanoparticles [43]. Peptides targeting Eph receptors can be employed for the selective delivery of chemotherapy in cancer cells overexpressing Eph receptors. Recently, Pasquale and co-workers showed that YSA PEGylated with lipid nanoparticles loaded with docetaxel showed improvements in terms of efficacy and toxicity when compared to the administration of the free drug, in a rat model of choroidal neovascularization [44].

The development of a peptidic ligand targeting Eph receptor is an active area of research and prompted the search for peptidomimetic agents. YSA was also the starting point for an extensive work made by Pellecchia and coworkers that led to the identification of novel peptidomimetic agonists with a nanomolar affinity for the EphA2 receptor and improved physicochemical properties. This was the case of dYNH peptide (Figure 1ii), in which replacement of two unnatural amino acids at the C-terminal portion and the substitution of L-Tyr with D-Tyr at the N-terminus allowed to achieve good plasma stability (10 times higher for dYNH than YSA), with a moderate loss of affinity for EphA2 [45]. This finding steered synthetic efforts toward a deep exploration of the role played by the N-terminal amino acid of EphA2 ligands. Therefore, employing an NMR-based binding assay using recombinant EphA2-LBD [46], researchers identified 123B9 (Figure 1iii), a peptidomimetic agent with an affinity comparable to the parent compound and an improved metabolic profile both in vitro and in vivo [45]. Subsequently, optimization of the C-terminal region of 123B9 structure identified two other peptidomimetics, 135E2 and 135G3, which resulted significantly more potent than the parent agents [47]. Moreover, the X-ray structure of 135E2 in complex with the EphA2 ligand-binding domain helped the structure-based optimization and subsequent identification of 135H11 (Figure 1iv), a peptidomimetic with a IC_50_ of 0.15 μM against EphA2 [47].

**Figure 2:**
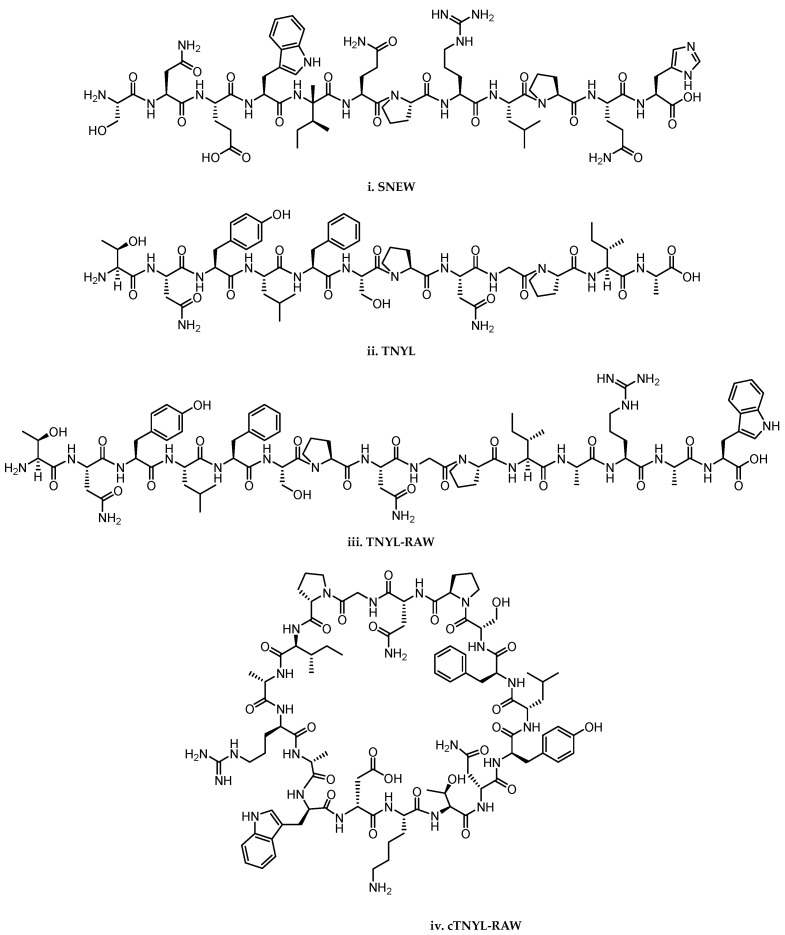
Structure of peptides and peptidomimetics antagonist of EphB2 and EphB4 receptors. **i**. SNEW; **ii**. TNYL; **iii**. TNYL-RAW; **iv**. cTNYL-RAW.

Phage display screenings were also performed to identify peptides selectively targeting EphB receptors, leading to SNEW (Figure 2i), a dodecameric peptide, able to bind EphB2 (K_D_ = 6 μM) while inhibiting ephrinB2 recruitment by EphB2 with an IC_50_ value of 15 μM [48]. Visual inspection of the SNEW-EphB2 crystallographic complex showed that SNEW adopts a different binding mode with respect to ephrinB2 ligand [49].

Another dodecameric peptide (TNYL—Figure 2ii) was identified as a weak inhibitor of the ephrinB2–EphB4 interaction (IC_50_ = 50–150 μM) [48]. Modifications of the C-terminal region TNYL led to TNYL-RAW (Figure 2iii) peptide, which displayed an inhibitory potency in the nanomolar range. The substantial increase in potency was ascribed to the ability of TNYL-RAW of undertaking additional productive interactions with EphB4-LBD. This hypothesis was validated by the resolution of the crystal structure of TNYL-RAW bounded to the EphB4 [50,51].

TNYL-RAW was characterized by short half-life in plasma and in cells due to its susceptibility to proteolytic degradation, thereby advancing attempts to increase its chemical stability were performed. Cyclization of TNYL-RAW provided cTNYL-RAW (Figure 2iv) which exhibits greatly increased stability in mouse plasma, without affecting the affinity for EphB4 [52]. Similar results were obtained through PEGylation or inclusion in gold nanosphere [52,53].

### 2.3. Bile Acids Derivatives: An Emerging Class of Small-Molecules Disrupting Eph-ephrin Interaction

An alternative strategy to interfere with signals transduced by the Eph-ephrin system is represented by small molecules targeting the protein-protein interaction (PPI) surface, and specifically by agents able to recognize the extracellular LBD of Eph receptors [54]. Small molecules able to interfere with the formation of a Eph-ephrin heterodimer can block both forward and reverse signals in cells with potential benefits in term of efficacy [10]. The presence in the LBD of Eph receptors of a large and solvent exposed cavity deputed to the recognition of ephrin ligands has discouraged drug discovery efforts in this area for many years. This negative thinking was reinforced by the finding that the activity displayed by the first class of small molecules reported to be active on the EphA2 receptor, i.e., 4-(2,5-dimethyl-CH-pyrrol-1-yl)-2-hydroxybenzoic acids, was actually due to an uncharacterized mixture of polymers of high molecular weight, accidentally generated after the exposure of these pyrrolyl-based compounds to air and light [55].

The resolution of X-ray structures of the LBD of many other Eph receptors in complex with ephrin ligands and the commitment of academic research groups favored the discovery of small molecules targeting this system. Most of the available complexes (i.e., EphA2-ephrin-A1 or EphB2-ephrin-A5) confirmed that the formation of the Eph-ephrin heterodimer was driven by the accommodation of the G-H loop present in ephrin ligands within a well-defined channel present in the LBD of the Eph receptors. Crucial for drug design was the identification of polar hot spots in the LBD of Eph receptors (such as Arg103 and Arg156 in EphA2), which later steered screening campaigns versus specific classes of compounds [41].

**Figure 3:**
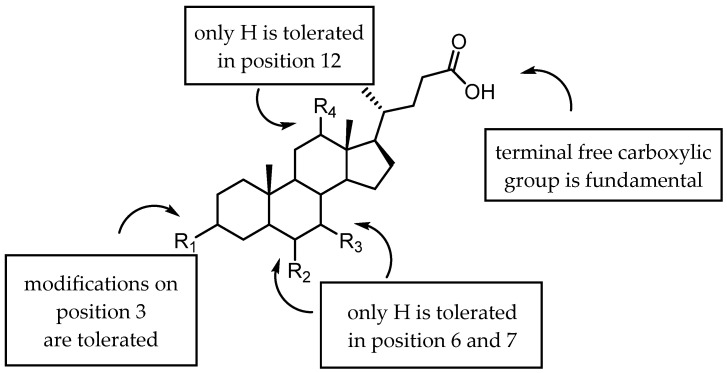
Graphical abstract of the general structure of bile acids antagonist of the EphA2 receptor and the identification of hot spots relevant for the activity.

The presence of accessible basic residues in the EphA2 LBD suggested that acid compounds might display some affinity for this receptor. A screening campaign of a chemical collection of acid compounds at the University of Parma allowed to identify lithocholic acid (LCA) as weak competitive antagonist of the EphA2, able to displace ephrin-A1 from EphA2 with a K_i_ = 49 µM [56]. Other bile acids, such as cholic (CA), deoxycholic (DCA), and chenodeoxycholic (CDCA), were unable to interfere with ephrin-A1 binding to EphA2. These preliminary results underlined the presence of tight structural requirements (summarized in Figure 3) for engaging the lipophilic channel present in the LBD of EphA2 [56].

A following investigation by Tognolini et al. in 2012 allowed expanding structure–activity relationships (SARs) around LCA [57]. In this work, it was confirmed that the introduction in position 6, 7, and 12 of the gonane core of LCA of polar substituents were not tolerated by EphA2, as shown by the lack of activity displayed by compounds **2**–**6** (Table 1). Conversely, modification of 3α-hydroxyl group appeared promising for the identification of more potent antagonists. Inversion of the absolute configuration of position 3 or removal of the 3α-hydroxyl group led to isolithocholic acid (**7**) or cholanic acid (**8**), which displayed *Ki* values of 25 and 5 μM, respectively. Other important information was inferred from the modification of the terminal carboxylic group of LCA. Compounds **9**–**11,** characterized by the presence of functional groups of lower acidity (**9** and **10**) or by an ester functionality (**11**), were inactive. Removal of an ionizable group (negatively charged at physiological pH) resulted in a detrimental effect on compound potency, suggesting that a terminal carboxylate was a fundamental pharmacophoric element for EphA2 binding [57].

**Figure 4:**
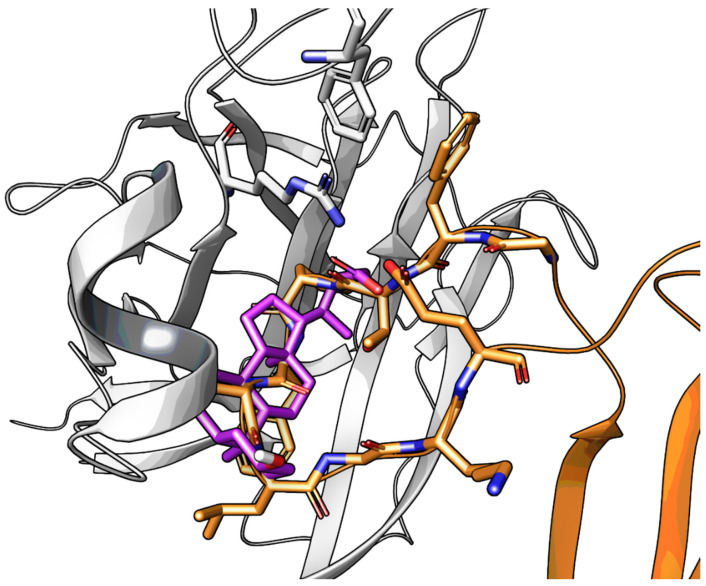
Visualization of the ephrin-A1 G-H loop (in orange) within the EphA2 receptor (white cartoon) and the docking pose of LCA (in purple). Arg103 and Phe108 of EphA2 are also displayed with a white stick representation.

Molecular modeling investigations suggested that LCA could bind the LBD of the EphA2 receptor occupying the same space of the ephrin-A1 G-H loop, by inserting its gonane scaffold into the hydrophobic channel present in the EphA2 receptor. According to this model, the 2-methylbutanoic acid chain of LCA could form a salt bridge with Arg103, mimicking the interaction of ephrin-A1 Glu119 [57]. The 3α-hydroxy group weakly interacted with Arg159 of EphA2 usually engaged in a hydrogen bond (H-bond) with Asp86 of ephrin-A1. Although other arrangements of LCA can be identified (i.e., with the gonane ring superposed on the G-H loop region but with the terminal carboxyl group of LCA forming salt bridge with Arg159), the binding mode reported in Figure 4 accounts to some extent for SAR data reported in Table 1, and more importantly, could be exploited for the design of novel Eph-ephrin antagonists.

Visual inspection of the EphA2-LCA complex generated by docking suggested that coupling of LCA with α-amino acids could give antagonists able to form a salt bridge with Arg103, while potentially forming additional productive interactions within the LBD of EphA2 [58]. The fair activity of glycolithocholic acid **12** (Table 2) suggested that conjugation was a promising strategy to identify better EphA2 ligands.

**Figure 5:**
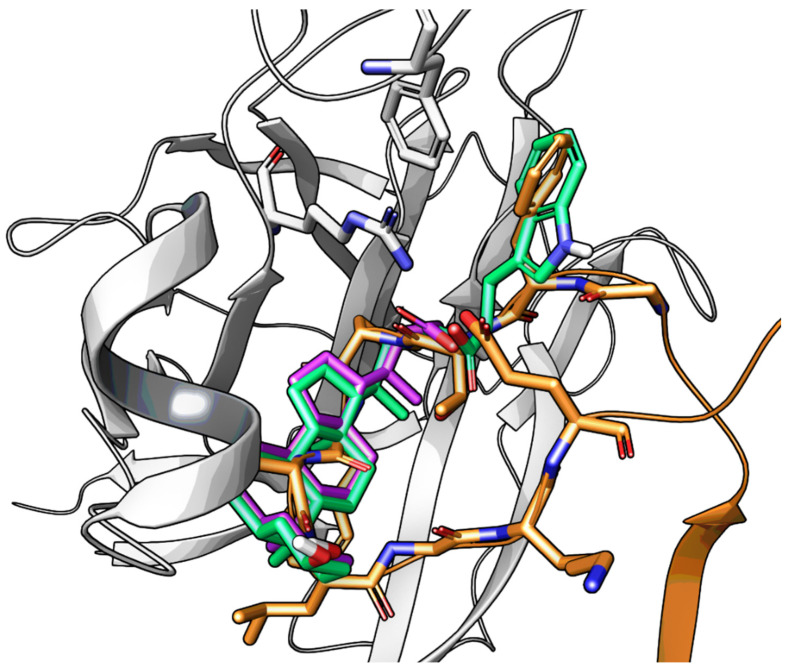
Docking of **UniPR126** (in green) within the EphA2 receptor (white cartoons, with Arg103 and Phe108 displayed by a stick representation). The indole moiety mimics the benzyl group of the Phe111 residue of the natural ligand ephrin-A1 (in orange). The free carboxylic group of **UniPR126** undertakes interactions with Arg103.

An experimental design, aimed at independently explore the impact of lipophilicity and steric hindrance on the inhibitory potency, identified a set of four α-amino acids (Ala, Asn, Ser, Val of both L- and D-series) to be conjugated with LCA for SAR evaluation. The resulting compounds (**14**–**21**, listed in the Table 2) were tested for their ability to displace biotinylated ephrin-A1 from EphA2 [59]. Compounds with a hydrophobic sidechain (Ala or Val, **14**–**17**) resulted more potent than those with polar chains (Ser or Asn, **18**–**21**). Other α-amino acids were conjugated to LCA giving compounds **22–31**, to further cover the space of lipophilic and steric properties. The introduction of amino acids with lipophilic side chains always led to active compounds. Notably, the introduction of aromatic substituents had a positive impact on the inhibitory potency. Phenylalanine conjugates **26** and **27** were ten times more potent than LCA. This trend was confirmed by the activity of tryptophan conjugates **30** and **31,** which were significantly more potent than LCA. With an IC_50_ value of 2.0 μM, the L-Trp conjugate (**30**, **UniPR126**) was the most potent antagonist of the series [59]. Noteworthy, the potency of **UniPR126** did depend on the stereochemistry of the α-amino acid, as D-Trp conjugate (**31**) was 10 times less potent than **UniPR126** [59]. This could be due to the accommodation of the indole moiety in a region of the EphA2 receptor which specifically recognizes aromatic substituents [41]. Docking of **UniPR126** within EphA2 receptor (Figure 5) supported this hypothesis as the indole ring of this antagonist occupied the same space of Phe111 of ephrin-A1 forming π–π interactions with Phe108 of EphA2 [59,60].

**Figure 6:**
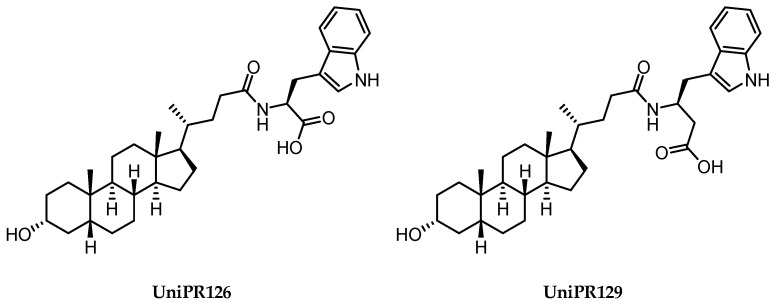
Structural representation of **UniPR126** and **UniPR129**.

Crucially, visual inspection of the EphA2−**UniPR126** complex also suggested that affinity for the EphA2 receptor could be increased by bringing the carboxylate group of UniPR126 closer to the guanidine group of Arg103 [61]. This working hypothesis prompted the synthesis of the L-β-Homo-Trp conjugate of LCA, **UniPR129** (Figure 6).

Pharmacological evaluation of **UniPR129** supported the improvement sought with the help of molecular modeling. This compound showed an IC_50_ of 0.9 μM for inhibition of ephrin-A1 binding to EphA2, a value two-fold lower than that reported for **UniPR126** (IC_50_ = 2 μM) [59,61]. In analogy to what was observed for **UniPR126**, **UniPR129** was able to interfere with the binding of ephrin ligands to all the cloned Eph receptors (i.e., EphA1-A8; EphB1-B4, EphB6) with similar potency, suggesting that this compound likely interacted with a conserved region of the LBD of the Eph receptors [61]. **UniPR129** emerged as useful pharmacological tool for in vitro experiments. **UniPR129** inhibited EphA2 autophosphorylation in human prostate cancer (PC3) cells, and it was able to modify prototypical phenotypes controlled by ephrin-A1 stimulation, including cell-rounding and migration. Additionally, at low micromolar concentration, **UniPR129** inhibited the ability of human umbilical vein endothelial cells (HUVECs) to form capillary-like tubes in vitro, proposing this compound as potential antiangiogenetic agents to be evaluated in vivo [61].

Nevertheless, **UniPR129** suffered from poor physicochemical properties which heavily hampered its bioavailability when orally administered to mice [62], and prompted investigators to further explore the SAR to yield antagonist suitable for in vivo administration.

In 2017, Incerti et al. reported an extended investigation on the SAR around UniPR129. The impact of the length of the linker connecting the terminal carboxylic acid to the amino group of the amino acid moiety on inhibitory potency was investigated (Table 3).

This analysis suggested that the β-alanine conjugate (**32**) had the optimal length as a further lengthening of the linker gave inactive compounds as observed in the cases of **33** and **34** [63]. Replacement of the carboxylic acid of **32** with a sulfonic acid (as for **35**) or its esterification (as for **36**) led to a significant loss of potency [63]. The presence of a negatively charged group in the lateral side chain of the amino acid moiety was beneficial for activity only when specific geometric requirements were satisfied.

Given these results, researchers focused their subsequent efforts on the synthesis and pharmacological evaluation of β-alanine conjugates of LCA bearing in β-position lipophilic sidechains of varying steric requirements (Table 4) [63].

Compounds possessing aliphatic chains (**37**–**39**) were generally less potent than the parent compound **32**. The introduction of a phenyl group with different spacers in the side chain resulted in an increase of the inhibitory potency, as in the case of compounds **40** and **41**. Only compound **42**, with a phenylethyl substituent, resulted inactive.

Important improvements were obtained with the introduction of larger aryl groups as side chain substituents. When the lateral chain was featured by a (i) indol-3-ylmethyl (**UniPR129**); (ii) α-naphthylmethyl (**43**); or (iii) benzo[b]thiophen-3-ylmethyl (**44**) substituent, a significant improvement in the inhibitory potency was observed, with the resulting compounds active in the low μM. **UniPR129** was the best compound of the series, and its higher potency compared to **43** and **44** could be ascribed to the presence of an indole N-H group, potentially able to form polar interactions within EphA2. This was somehow supported by the significant drop of activity displayed by diastereomer of **UniPR129** (compound **45**), in which the absolute configuration of the β carbon was inverted [63]. To better understand the possible interactions formed by **UniPR129** within EphA2, molecular dynamics (MD) simulations were performed. These simulations supported the formation of a H-bond occurring between the N-H of the indole ring of **UniPR129** and the carboxylate group of Asp53, giving a model-based explanation to the observed SAR [63].

The same MD simulations suggested that the 3α-hydroxyl group of **UniPR129** was an important anchor point for EphA2 binding, being able to form a H-bond with the side chain of Asn57 and/or with Ile58 backbone [63]. This computational suggestion opened a new route for further investigations. In this scenario, a SAR evaluation directed towards position 3 of **UniPR129** (Table 5) was performed with the two-fold aim of improving inhibitory potency and modulate physicochemical properties.

A focused set of modifications at position 3 of **UniPR129** was planned to confirm if the presence of a substituent able form a H-bond with EphA2 was critical for the activity. To this end, the 3α-hydroxyl of **UniPR129** was replaced by a 3β-hydroxyl (**46**), by a hydrogen (**47**), or oxidized to a 3-keto functional group (**48**). A significant loss in the inhibitory potency was observed for these three analogues [63].

These results prompted the insertion of other groups able to form productive polar interactions, including H-bonds, within EphA2. Replacement of the 3α-hydroxyl of **UniPR129** with a 3-hydroxyimino led to compound **49,** which resulted rather potent at preventing ephrin-A1 binding to EphA2. Crucially, compound **51** (also known as **UniPR502**), a 3α-carbamoyloxy derivative, resulted slightly more potent than **UniPR129** showing an IC_50_ value of 0.8 μM. Methylation of **49** and inversion of the C3 configuration of **UniPR502** gave compounds **50** and **52**, respectively which were less potent than their cognate analogues by nearly five-fold [64].

The slightly improvement of the inhibitory potency obtained with **UniPR502**, led to a follow-up exploration. Efforts were thus taken to exploit the carbamoyloxy functionality and to introduce a point for chemical diversification into the 5β-cholan-24-oic acid scaffold of **UniPR502**, to search for better EphA2 antagonists.

The nitrogen atom of the 3α-carbamoyloxy was functionalized (Table 6) with different substituents [64], while preserving the possibility to form a H-bond with EphA2 receptor. The ethyl, butyl and hexyl carbamate derivatives, **53** (**UniPR505**), **54** and **55**, showed IC_50_ values in line with that of **UniPR502**, suggesting that alkyl chains of moderate size were tolerated by EphA2 receptor. The further lengthening of the alkyl chain was detrimental for activity as in the case of octyl derivative **56** that displayed a marked decrease of the inhibitory potency (IC_50_ = 15 µM) [64]. Finally, the introduction of branched or cyclic substituents gave compounds (i.e., **57**, **58**, **59**) fair potency but always lower than that displayed by the reference EphA2 antagonist **UniPR502**.

Compound **53** (**UniPR505**) emerged as one of the most interesting compounds of this series and it was the subject of an intense biochemical characterization. **UniPR505** demonstrated to act as a competitive and reversible antagonist of the EphA2 receptor, displaying a Ki of 0.3 μM in ELISA binding assay. When tested on PC3 cells, **UniPR505** dose-dependently inhibited EphA2 phosphorylation in PC3 cells with an IC_50_ of 1.5 μM, very close to the inhibitory activity showed in the cell-free displacement assay (IC_50_ of 0.95 μM) [64]. When tested on endothelial cells (HUVECs), **UniPR505** was able to dose-dependently inhibit angiogenesis with an IC_50_ value lower 3 μM, resulting a better antagonist than **UniPR129**. This prompted the use of **UniPR505** in an in vivo model of neovascularization such as the chick chorioallantoic membrane (CAM) system. In the CAM model, **UniPR505** dramatically reduced the growth of blood vessels in the presence of angiogenic growth factors [64]. Finally, differently from what reported for 3α-hydroxy-5β-cholan-24-oyl amino acid conjugates (including **UniPR126** and **UniPR129**, able to engage all the Eph receptors with comparable inhibitory potency), **UniPR505** demonstrated a preference for the EphA2, since it spared some Eph receptor subtypes i.e., EphA1, EphA7, EphB1, EphB4, and EphB6. **UniPR505** emerged as one of the most interesting Eph-ephrin antagonist with a moderate selectivity for the EphA2 receptor, and physicochemical properties compatible with its use in vivo [64].

#### 2.3.1. A New Scaffold for EphA2 Receptor Antagonists: Discovery of 3β-Hydroxy-Δ^5^-Cholenic Acid

**Figure 7:**
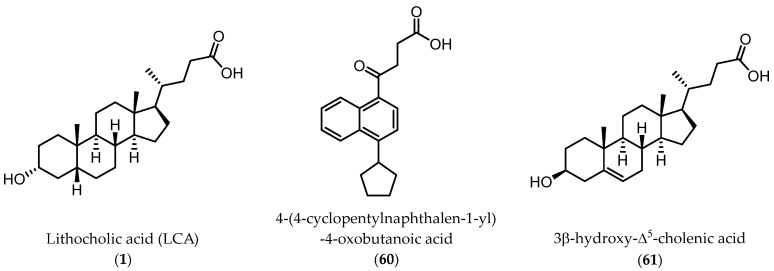
Chemical structures of compound **60** and **61** identified as analogues of **1** by virtual screening of a library of commercially available compounds.

Despite the positive results and the improvements with amino acid conjugates of LCA, other strategies were taken to discover new chemotypes working on the EphA2 receptor. A virtual screening campaign performed at University of Parma identified, 4-(4-cyclopentylnaphthalen-1-yl)-4-oxobutanoic acid (**60**) and 3β-hydroxy-Δ^5^-cholenic acid (**61**) (Figure 7) as inhibitors of the EphA2−ephrin-A1 interaction [65].

While the 3β-hydroxy-Δ^5^-cholenic acid (**61**) showed an inhibitory activity comparable to that of LCA, the geometry of its steroidal scaffold, resembling that of a 5α-gonane nucleus in contrast to the 5β-gonane of LCA, had the potential to ensure to **61** itself a better selectivity profile. Intriguingly, compound **61** did not engage classical targets of bile acids, including nuclear receptors (FXR, PXR) and G-protein coupled receptors [65], proposing it as new scaffold for the generation of new Eph-ephrin antagonists.

**Figure 8:**
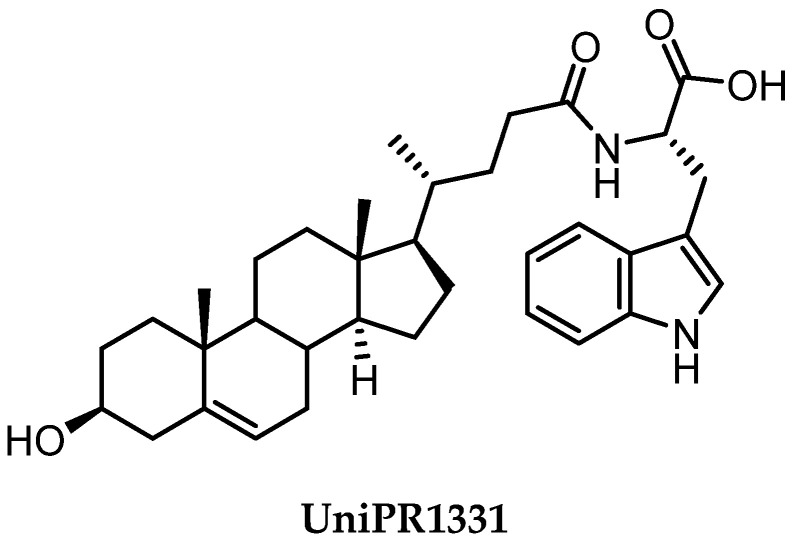
Structure of UniPR1331.

Conjugation of **60** with L-Trp led to an inactive compound, while conjugation of **61** with the same amino acid generated a new EphA2 antagonist named, **UniPR1331** (Figure 8), featured by low micromolar affinity (IC_50_ value of 3.3 μM, vide infra) for this receptor. This result paralleled what observed for LCA, in which conjugation with L-Trp guaranteed a significant improvement in the inhibitory potency on EphA2-ephrin-A1 system, with the resulting compound (**UniPR126,** compound **30**) displaying an IC_50_ value of 2.0 μM.

Even though **UniPR126** and **UniPR1331** share a high 2D similarity, these two compounds significantly differ in their 3D geometry. The bile acid moiety of **UniPR126** has a bent shape due to a *cis*-junction connecting ring A and B of the 5β-gonane scaffold, while Δ^5^-cholenic acid moiety of **UniPR1331** has an extended structure due to a *trans*-like junction between A and B ring of the gonane nucleus arising from the presence of a double bond between C5 and C6 atoms.

**Figure 9:**
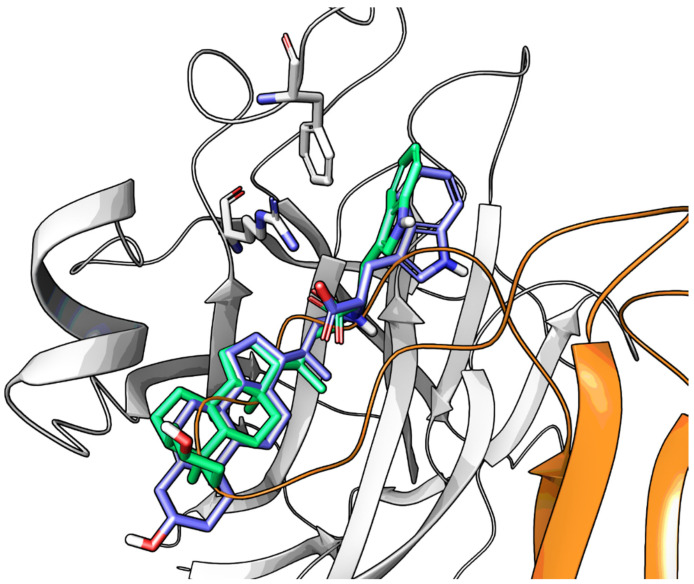
Comparison between the docking poses of **UniPR126** (in green) and **UniPR1331** (in blue) within EphA2 receptor (white cartoons). The A ring of the steroid portion of **UniPR1331** assumes a different orientation within the receptor due to the presence of a double bond between C5 and C6 of the gonane scaffold.

Despite this structural difference, both compounds adopted a similar binding mode at the level of the L-Trp moiety (Figure 9), suggesting that the SAR at the level of amino acidic portion should be somehow transferable from LCA conjugates to 3β-hydroxy-Δ^5^-cholenic conjugates [66].

#### 2.3.2. Amino Acid Conjugates of 3β-Hydroxy-Δ^5^-Cholenic Acid as EphA2 Antagonists 

The synthesis of a new set of compounds based on the 3β-hydroxy-Δ^5^-cholenic acid structure was thus performed. The 3β-hydroxy-Δ^5^-cholenic acid nucleus was conjugated to α-amino acids of small and large sizes, as reported in Table 7.

Activity data obtained from the EphA2-ephrin-A1 displacement assays were somehow in line with the SAR drawn for LCA-derivatives. When **61** was conjugated with amino acids bearing aliphatic side chains, the resulting compounds **62**–**64** were inactive. Conjugation with aromatic amino acids gave inactive (L-Phe **65**) or weakly active (L-O-phenylserine **66**) derivatives [66]. However, when L-Trp was coupled with 3β-hydroxy-Δ^5^-cholenic acid **61**, the resulting conjugate **UniPR1331** emerged as a fair EphA2 antagonist able to displace ephrin-A1 from EphA2 at low micromolar concentration (IC_50_ value of 3.5 μM). Conjugation of cholenic acid with D-Trp gave compound **67,** which lacked activity on the EphA2 receptor [66]. This suggested that the indole ring of the L-Trp of **UniPR1331** was involved in specific interactions with EphA2 and it could represent a starting point for an informative SAR exploration. Modification of the indole N-H group by methylation (compound **68**) or replacement of the indole itself with a naphthalene group (compound **69**), led to antagonists with potency comparable to that of **UniPR1331** (IC_50_ of 5.0 and 4.1 μM for **68** and **69,** respectively), indicating that the indole N-H group of **UniPR1331** was not involved in crucial polar interactions with EphA2.

The indole ring of **UniPR1331** was further exploited for SAR investigations by inserting in position 5 a set of substituents with different stereoelectronic properties (see compounds **70**–**73)**. Regardless of the electronic properties, only substituents featuring a low steric hindrance (-F, -OH) were tolerated by EphA2. Only compounds **70** and **72** displayed a potency value close to that of **UniPR1331**.

Finally, the importance of the two polar ends of **UniPR1331** for EphA2 inhibition was investigated with the synthesis of additional compounds. When the terminal carboxylic acid group of **UniPR1331** was methylated, the resulting methyl ester **74** was inactive indicating that a free carboxylic group was essential for activity also in the case of the 3β-hydroxy-Δ^5^-cholenic acid series. Removal of a H-bond donor group by acetylation of the 3β-hydroxyl group had a detrimental effect on the inhibitory potency as indicated by lack of potency of compound **76**.

**Figure 10:**
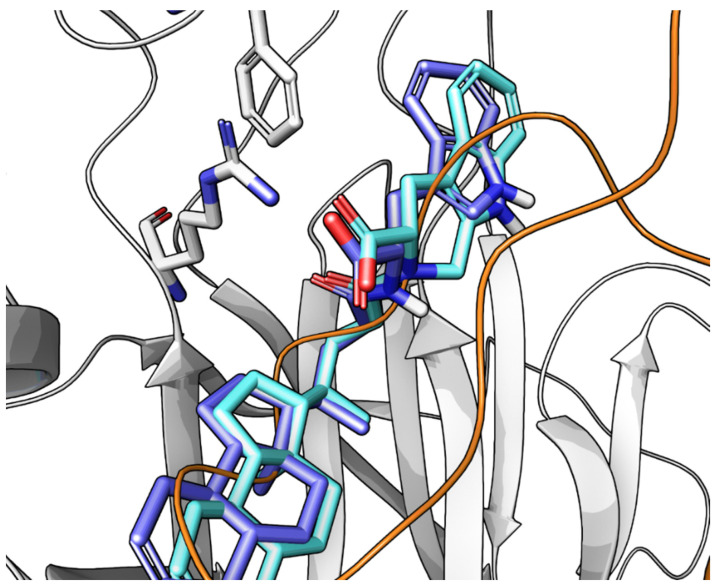
Magnification of the terminal portion of the docking poses of **UniPR1331** (blue) and compound **77** (light blue). The indole ring assumes the same position and orientation inside the receptor.

On the base of SAR data and thanks to docking simulations alternative binding modes for **UniPR1331** within EphA2 were proposed. These differed only for the orientation of the L-Trp moiety in the LBD of EphA2. To identify the most-likely binding pose for **UniPR1331**, a conformationally restricted analogue, in which position 2 of the indole ring was connected to the nitrogen of the amino acid by a methylene linker, was synthetized and tested. The resulting compound **77** displayed an IC_50_ value of 6.3 μM [66], close to that of **UniPR1331**. The orientation assumed by the indole ring of compound **77** matched only one of the proposed docking poses of **UniPR1331** (Figure 10), in which the indole ring was able to form a π-π interaction with Phe108 of EphA2. Overall, this confirmed that L-Trp improved inhibitory potency over other conjugates thanks to specific interactions with EphA2 [66].

#### 2.3.3. UniPR1331: An Emerging Eph-ephrin Antagonist for In Vivo Study

**UniPR1331** became the subject of an intensive biochemical characterization aimed at clarifying its mechanism of action, its selectivity versus other Eph receptors and versus biological targets modulated by bile acids.

Surface plasmon resonance investigations showed that **UniPR1331** binds EphA2 in a concentration-dependent and saturable manner, with 1:1 stoichiometry, and an affinity constant (K_D_) value of 3.3 μM [66]. This confirmed that the ability of this compound to competitively displaces ephrin-A1 from EphA2 relies on the ability of **UniPR1331** to bind the same site on EphA2 recognized by ephrin-A1.

By strict analogy to what was observed in the case of bile acid derivatives **UniPR126** and **UniPR129**, **UniPR1331** also inhibited ephrin binding to all members of the Eph receptor family with low micromolar potency. **UniPR1331** displayed excellent selectivity with respect to molecular targets activated by bile acids as it did not interfere with the activity of TGR5, FXR, and PXR receptors [66].

Experiments in cells showed that **UniPR1331** inhibited phosphorylation of EphA2 in prostate cancer (PC3) cells without directly interfering with the kinase domain. In endothelial cells (HUVEC model), **UniPR1331** blocked angiogenesis in a concentration-dependent manner with an IC_50_ value of 2.9 μM [66]. These promising in vitro data proposed **UniPR1331** as reference Eph-ephrin antagonist and prompted researchers to evaluate if **UniPR1331** could have also better pharmacokinetics (PK) properties than the bile-acid ligand **UniPR129**.

Further investigations showed that **UniPR1331** had a fair PK profile. In fact, **UniPR1331** presented a C_max_, after a single administration (30 mg/kg, os), at 30 min of 1.4 μM, a hundred-fold higher than what observed for **UniPR129** [66]. The improvement was also significant in the systemic exposure, represented by the area under the curve (AUC_0-t_) with a value of 573.1 ng/mL h, compared to 21.2 ng/mL h displayed by **UniPR129**. The improvement was likely due to a different steroidal core that guaranteed a better oral bioavailability, with **UniPR1331** less susceptible to liver metabolism than **UniPR129** [66].

Despite the improvement of the PK profile, **UniPR1331** showed a rapid decline in plasma after oral administration. To better understand the in vivo behavior of this compound, metabolic stability assays were performed to evaluate phase I and phase II biotransformations [67]. Characterization of **UniPR1331** metabolites was performed using high performance liquid chromatography coupled to tandem mass spectrometry (HPLC-ESI-MS/MS) and high-resolution mass spectrometry (HPLC-ESI-HR-MS). In vitro metabolism data did not account for the short biological half-life observed in mice following oral administration of **UniPR1331**. Moreover, the in vitro metabolic profiling did not match with the in vivo profile of **UniPR1331** metabolites, except for a keto analogue at C3 position. A different metabolite was detected after oral administration, which was never identified in vitro. Intriguingly, exposure in vitro to mice fecal material of **UniPR1331** produced the very same main metabolite observed in vivo, supporting a major role of gut microbiota for the metabolism of **UniPR1331** [67].

Despite a suboptimal PK profile, **UniPR1331** displayed promising antitumoral activity when tested in vivo by the oral route. Extended work performed in collaboration with the group of Festuccia at the University of L’Aquila allowed to investigate the antitumoral activity of **UniPR1331** on in vivo models of glioblastoma multiforme (GBM). **UniPR1331** (administered per *os* at 30 mg/kg 5 days per week) dramatically reduced GBM growth in two distinct xenograft models (based on U87MG and U251MG cells), leading to a significant delay in the time to progression of the disease [68].

Biochemical analysis of the GBM indicated that this marked antitumoral response was due to a dramatic blockage of angiogenesis and vasculomimicry obtained through the inhibition of the activity of the Eph-ephrin system and, to some extent, to the inhibition of the VEGF–VEGFR axis, as recently suggested by throughout work of Rusnati and collaborators [69].

**Figure 11:**
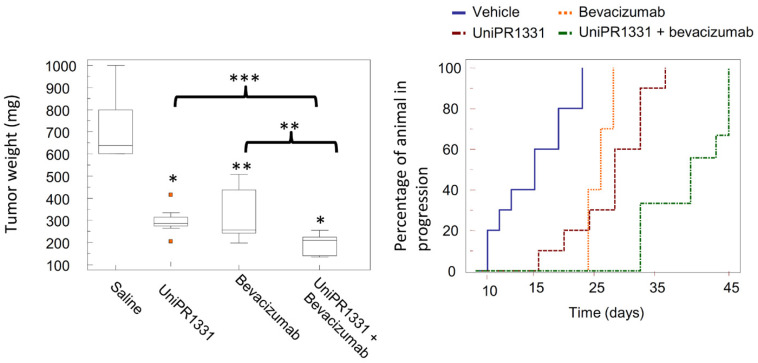
Effect of **UniPR1331** on GBM growth (U87MG cells)**.** Effect on tumor weight in GBM xenograft mice model. Kaplan-Meier curves for **UniPR1331** (red dotted line), bevacizumab (orange dotted line) or a combination of **UniPR1331** with bevacizumab (green dotted line). Blue line represents the vehicle. *p* values <0.05 were considered statistically significant. * <0.05; ** <0.01; *** <0.001.

Combination of **UniPR1331** with the anti-VEGF monoclonal antibody bevacizumab dramatically reduced vasculomimicry with appearance of large avascular tumor areas in U87MG tumors with beneficial effects for treated mice (Figure 11) [68]. The activity of **UniPR1331** (alone or in combination with bevacizumab) was also evaluated on orthotopic models generated from patient-derived GBM stem cells injected in the brains of nude mice. In this set of experiments, a significant increase of the overall survival was observed in treated animals, confirming the ability of **UniPR1331** to act as an effective anti-GBM agent.

### 2.4. Kinase Inhibitors

Another approach to interfere with the Eph-ephrin exploits the clinically validated approach of tyrosine-kinase inhibitors (TKI), directed towards the ATP binding site of the kinase domain of Eph receptors with small molecules. Advances in this field have been recently discussed in the literature [70]. These inhibitors were further exploited as biological tools for the development of radiotracers, to investigate the role and impact of the Eph-ephrin system in cancer, as recently described by Neuber et al. [71].

## 3. Conclusions and Perspective

Clinical evidence has demonstrated the involvement of the Eph-ephrin system in pathological conditions, especially in cancer insurgence and progression. Targeting this signaling pathway has emerged as a possible strategy to tackle cancers in which the activity or expression of Eph receptors or ephrin ligands is abnormal compared to healthy tissues. Several pharmacological approaches have been proposed and attempted in last 20 years. Kinase inhibitors have been used to target Eph-ephrin system, but while being able to block Eph receptor phosphorylation (forward signal), they are unable to inhibit the activation of ephrins (reverse signal). Moreover, their limited selectivity, due to interaction with several members of the kinome, has been observed [72].

A promising strategy, represented by small molecules able to interfere with the formation of the Eph-ephrin signaling system through the selective targeting of the extracellular LBD of the Eph receptors, has recently emerged. Compounds with such a mechanism of action (protein-protein interaction inhibition, PPI-i) can indeed simultaneously inhibit the forward and the reverse signaling in contiguous cells with potential therapeutic benefits, surpassing in terms of selectivity Eph kinase inhibitors.

Different classes of small molecules have been reported in the literature in the last decade, most of them designed around the structure of the secondary bile acid, lithocholic acid (LCA), previously reported to be a physiological agonist of TGR5 and FXR receptors. By a combined effort merging structural data, computer-aided drug design and pharmacological assays, researchers have exploited the weak activity of LCA on EphA2 receptor to devise potent compounds, culminating in the L-Trp and L-β-Homo-Trp conjugates of LCA, known as **UniPR126** and **UniPR129**. These compounds displayed fair potency in vitro and SAR investigations around their structures allowed to uncover key information: (a) the importance of their terminal carboxylic group, needed to bind Arg103 of the EphA2; (b) the impossibility of replacing the steroid portion with less hydrophobic scaffolds; (c) the importance of a polar group in position 3, capable of forming H-bonds with the LBD of the Eph receptor.

To further advance this class of compounds, aiming to eliminate potential off-targets, the search for chemotype alternatives to LCA led to the identification of 3β-hydroxy-Δ^5^-cholenic acid as a novel scaffold to be exploited for the synthesis of new Eph-ephrin antagonists. A medicinal chemistry optimization prompted the discovery of **UniPR1331**, the L-Trp conjugate of 3β-hydroxy-Δ^5^-cholenic acid, which overcame most of the specific limitations observed with LCA-based compounds, such as selectivity over TGR5 and FXR receptors, and poor bioavailability [66]. 

**UniPR1331** was reported to have significant in vivo antiangiogenic properties and a remarkable anti-tumor activity in animal models against GBM. **UniPR1331** increased the disease-free survival in both xenograft and orthotopic mice models of GBM [68]. Thus, **UniPR1331** has emerged as a promising tool compound to be considered as a starting point for the generation of Eph-ephrin antagonists, featuring higher potency and improved metabolic stability.

## Figures and Tables

**Table 1:** *K_i_* values of LCA derivatives measured by an ELISA binding assay.

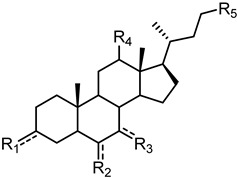						
Cpd.	R_1_	R_2_	R_3_	R_4_	R_S_	K_i_ (μM)
**1 (LCA)**	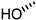	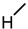	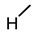	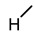	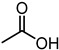	49
**2 (CA)**	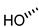	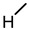	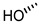	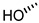	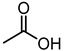	>100
**3 (DCA)**	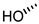	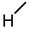	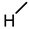	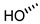	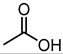	>100
**4 (CDCA)**	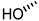	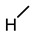	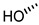	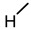	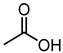	>100
**5**	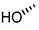	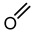	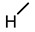	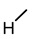	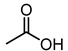	>100
**6**	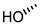	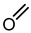	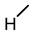	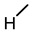	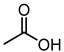	>100
**7**	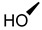	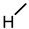	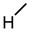	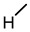	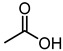	25
**8**	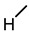	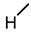	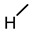	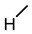	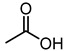	5.1
**9**	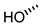	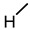	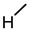	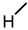	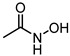	>100
**10**	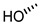	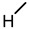	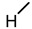	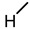	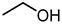	>100
**11**	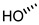	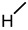	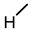	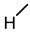	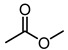	>100

**Table 2:** IC_50_ values for amino acid conjugates of LCA measured by ELISA binding assay.

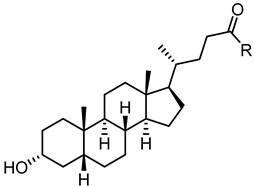					
Cpd.	R	IC_50_ (μM)	Cpd.	R	IC_50_ (μM)
**1 (LCA)**	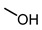	57	**22**	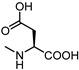	>100
**12**	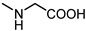	49	**23**	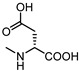	>100
**13**	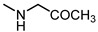	< 300	**24**	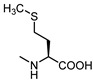	28
**14**	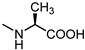	20	**25**	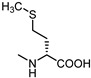	28
**15**	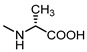	31	**26**	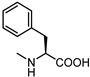	6.6
**16**	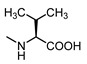	24	**27**	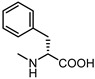	7.5
**17**	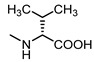	17	**28**	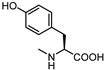	50
**18**	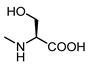	33	**29**	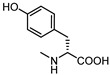	100
**19**	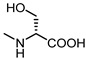	60	**30**	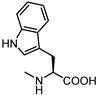	2.0
**20**	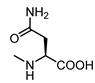	>100	**31**	** 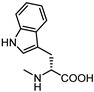 **	20
**21**	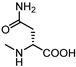	>100			

**Table 3:** IC_50_ values of LCA derivatives coupled with linear natural and unnatural amino acids.

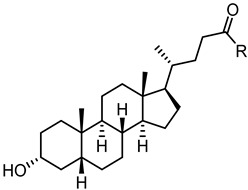		
Cpd.	R	IC_50_ (μM)
**LCA**	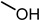	57
**12**	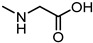	49
**32**	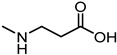	29
**33**	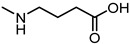	inactive
**34**	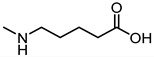	Inactive
**35**	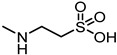	72
**36**	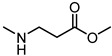	inactive

**Table 4:** IC_50_ Values of β-homo-amino acid conjugates of LCA.

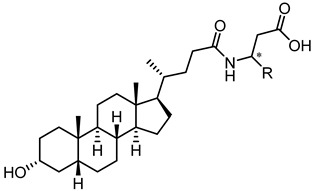		
Cpd.	R	IC_50_ (μM)
**32**	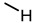	29
**37**	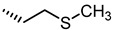	83
**38**	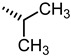	10
**39**	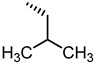	Inactive
**40**	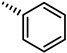	14
**41**	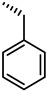	18
**42**	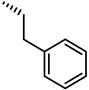	Inactive
**43**	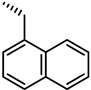	3.9
**44**	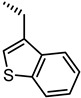	1.8
**UniPR129**	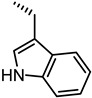	0.95
**45**	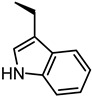	26

**Table 5:** IC_50_ of 3-Substituted 5β-Cholan-24-oyl-L-β-homo-tryptophan derivatives.

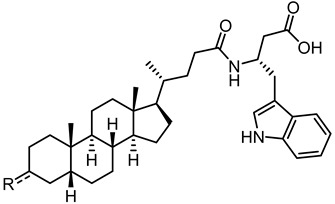		
Cpd.	R	IC_50_ (μM)
**UniPR129**	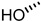	0.95
**46**	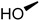	17
**47**	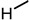	28
**48**	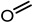	28
**49**	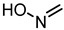	3.1
**50**	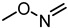	13
**51**	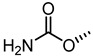	0.8
**52**	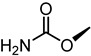	5.2

**Table 6:** IC_50_ values for 5β-cholan-24-oyl-L-β-homo-tryptophan derivatives.

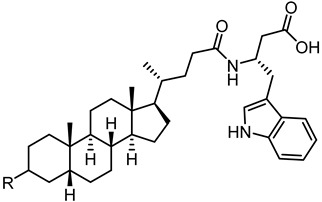		
Cpd.	R	IC_50_ (μM)
**UniPR129**	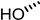	0.91
**UniPR502**	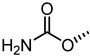	0.8
**53 ** **(UniPR505)**	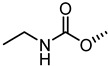	0.95
**54**	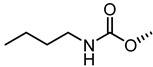	1.1
**55**	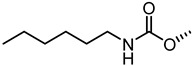	1.5
**56**	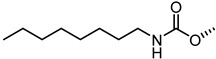	15
**57**	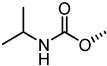	7.1
**58**	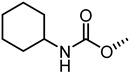	1.2
**59**	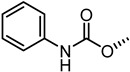	5.5

**Table 7:** IC_50_ values for compounds **62**–**77** tested in the EphA2-ephrin-A1 ELISA assay.

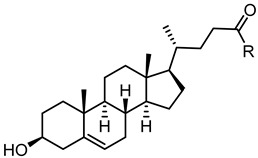					
Cpd.	R	IC_50_ (μM)	Cpd.	R	IC_50_ (μM)
**61**	** 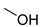 **	40	**69**	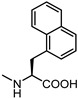	4.1
**62**	** 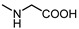 **	inactive	**70**	** 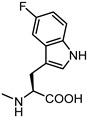 **	8.9
**63**	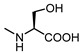	>100	**71**	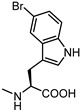	22
**64**	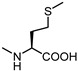	inactive	**72**	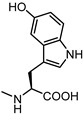	6.0
**65**	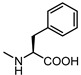	>100	**73**	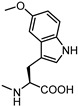	47
**66**	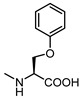	50	**74**	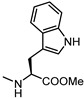	inactive
**UniPR** **1331**	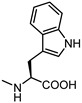	3.5	**75**	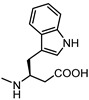	4.2
**67**	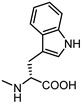	>100	**76**	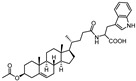	89
**68**	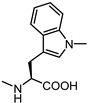	5.0	**77**	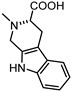	6.3

## Data Availability

Date sharing is not applicable.
